# Perioperative Outcomes of Total Vaginal Hysterectomy Versus Total Abdominal Hysterectomy: A Study of the Enlarged Uterus with Uterine Prolapse from Romania

**DOI:** 10.3390/medicina62020321

**Published:** 2026-02-04

**Authors:** Mihnea Nicodin, Laura Nicodin-Tigoianu, Anca Popescu, Mariam Dalaty, Diana Badiu, Lucian Cristian Petcu, Ovidiu Nicodin, Cristian Delcea, Nicolae Suciu

**Affiliations:** 1 Doctoral School of Medicine, “Carol Davila” University of Medicine and Pharmacy, 8, Eroii Sanit Blvd., 050474 Bucharest, Romania; 2 “Carol Davila” Central Military Emergency University Hospital, 134, Plevnei Street, Sector 1, 010825 Bucharest, Romania; 3 Faculty of Medicine, Titu Maiorescu University, 67 A, Gheorghe Petrascu Street, Sector 3, 031593 Bucharest, Romania; 4 Alessandrescu Rusescu National Institute for the Health of Mother and Child, 38-54, Gh. Polizu Street, 020395 Bucharest, Romania; 5 Doctoral School of Medicine and Pharmacy, “George Emil Palade” University of Medicine, Pharmacy, Science, and Technology of Targu Mures, 38, Gh. Marinescu Street, 540142 Targu Mures, Romania; 6 Faculty of Medicine, “Ovidius” University from Constanta, 1, University Str., 900470 Constanta, Romania; 7 Doctoral School of Medicine, “Ovidius” University from Constanta, 1, University Str., 900470 Constanta, Romania; 8 Multidisciplinary Doctoral School, Vasile Goldis Western University of Arad, 94, Bvd. Revolution, 310025 Arad, Romania

**Keywords:** vaginal hysterectomy, uterine prolapse, enlarged uterus, blood loss, hospital stay

## Abstract

*Background and Objectives:* Total vaginal hysterectomy (TVH) has been performed quite often for a uterus with prolapse, although less used than total abdominal hysterectomy (TAH). The purpose of this study was to compare the perioperative outcomes of patients who underwent either TVH or TAH for a uterus with a weight between 250 and 300 g and uterine prolapse (UP). *Materials and Methods:* In this retrospective study, 180 hysterectomies were planned for women with UP between 2020 and 2024 in a tertiary center in Romania. Patients were diagnosed based on clinical symptomatology and transabdominal ultrasound. All hysterectomies were performed by the same surgeon and were divided into two groups: TVH group (*n* = 90) and TAH group (*n* = 90). Patients’ characteristics like age, uterine weight, body mass index (BMI), parity, operative time, intra-operative blood loss, hospital stay, medical history, surgical history, intra- and post-operative complications, and adhesions were evaluated. *Results:* No significant differences were found between groups in terms of mean age, uterine weight, BMI, or parity. TVH was associated with significantly shorter operative time, lower intra-operative blood loss, and reduced hospital stay compared to TAH (*p* < 0.001). Both medical and surgical histories were more common in the TAH group compared with the TVH group. However, post-operative complications were slightly more frequent in the TAH group (9.99% vs. 3.33%), as were adhesions (33.33% vs. 13.33%). Uterine hemisection, tactical myomectomy, or morcellation were used in most cases to obtain a reduction in uterine size for the TVH group (81.11%). *Conclusions:* Our results showed that shorter operating time, lower intra-operative blood loss, and reduced hospital stay support the use of TVH in the case of an enlarged uterus with UP. The present study showed that all patients requiring hysterectomy for such conditions can be offered TVH, which could represent a better therapy option.

## 1. Introduction

Uterine prolapse (UP) represents the displacement of the uterus from its normal position. These displacements have been graded on a scale from zero to four, which increases with increasing severity of the prolapse. Zero grade refers to no prolapse, and four refers to total prolapse [1]. The main symptoms sustained by patients are local, involving urinary, bowel, or sexual issues. The identified etiological factors include congenital weakness of tissues, parity, aging, and lifestyle [2]. Depending on the prolapse grade, the treatment could be conservative or surgical [1]. In this context, total vaginal hysterectomy (TVH) was initially used for UP, although its indications now increased.

TVH is considered less invasive than total abdominal hysterectomy (TAH), being preferred for its many advantages over TAH [3,4]. Indications for TVH or TAH include leiomyomas, endometriosis, prolapse, pelvic inflammatory disease, endometrial hyperplasia, abnormal uterine bleeding, menorrhagia, dysmenorrhea or pelvic pain, postpartum hemorrhage, endometrial hyperplasia, or malignancies [5,6]. Depending on the different conditions of patients, the type of hysterectomy is selected. These include pelvic anatomy, adnexal disease, gastrointestinal complaints, uro-gynecological issues, heart disease, body mass index (BMI), parity or surgical interventions, and most importantly, the uterine size [7]. TVH still represents a method of choice compared to TAH, presenting fewer complications [8], being also a common surgical procedure, second only to cesarean delivery [8]. Among the known advantages are the following: it enables removal of a normal or slightly larger-than-normal uterus, usually having a faster recovery, tending to cause less pain during recovery, without leaving scars on the abdomen [9]. The most important post-operative complications for both hysterectomies are hemorrhage (0.2–2%) [10], followed by post-operative febrile morbidity and infection in 10% [6], bladder injury in 2.9%, and ureteral injuries in approximately 0–1.8% of cases [11]. However, damage to the bowel is quite uncommon, particularly in TVH [12], and almost 80% of injuries occur at the junction of the ureter and uterine artery [13].

Approximately 38.5% of women aged 45 years and older have an anatomical UP that could require surgical repair, with a recurrence rate of 29% [14,15]. However, both types of hysterectomy represent a proven risk factor for UP, knowing that surgery itself is hypothesized to contribute to damage of the supportive tissue [16,17]. Although uterosacral ligaments have an important role in pelvic floor support, their cause or how they are affected is still debated. Notably, it is supposed that the uterosacral ligaments may be damaged less during TVH than during the open abdominal approach. Whether this leads to a different prevalence of UP later in life is yet not well known [18].

The aim of this study was to compare the perioperative outcomes of patients in our hospital population, undergoing either TVH or TAH for a uterus with a weight between 250 and 300 g, with UP.

## 2. Materials and Methods

### 2.1. Study Design and Participants

A total of 180 patients were recruited retrospectively, over a four-year period between 1 February 2020 and 30 November 2024, among patients scheduled for hysterectomy with bilateral adnexectomy for the uterus with UP in the Department of Gynecology from “Carol Davila” Central Military Emergency University Hospital, Bucharest, a tertiary center in Romania. The patients were all in postmenopausal state and scheduled for surgical interventions because of the irreducible prolapse after several years of failed conservative treatment (i.e., pessaries). The patients were divided into two groups as follows: TVH group (*n* = 90 women) and TAH group (*n* = 90 women), providing a strong statistical power and allowing for reliable comparisons.

Data recorded included age, uterine weight, BMI, parity, operative time, intra-operative blood loss, hospital stay, medical history, surgical history, intra- and post-operative complications, and adhesions. Patients who were admitted under the indications of chronic pelvic pain, cervical dysplasia, pelvic inflammatory disease, or any malignancy were excluded from the study. All patients who met the inclusion criteria (i.e., patients with UP, uterine weight between 250 and 300 g, and/or other pathologies like fibroids or adenomyosis) were enrolled in the study. Patients were diagnosed based on clinical symptomatology (i.e., pelvic pressure or heaviness, seeing or feeling the cervix bulge from the vagina, lower back pain, or constipation) and transabdominal findings. All patients were scanned by one operator on the day prior to the operation, using an ultrasound machine with 3.5 mHz transabdominal probes. Two views were obtained, longitudinal and transverse. In the longitudinal view, the longitudinal measurement was taken from the highest fundal point in the midline to the corresponding midline cervico-uterine junction (uterine length). In the same view, the anteroposterior measurement (uterine depth) was taken at 90° to this plane, at the widest fundal dimension. In the transverse view, the largest transverse dimension (uterine width) was measured at the insertion of the fallopian tubes into the uterine fundus. The following day, during the hysterectomy, the uterus was immediately measured after its removal. The three maximal dimensions of length, depth, and width were obtained by using a caliper and centimeter scale on the same axis as that described for the ultrasonographic measurements. Uterine volume was calculated using the prolate ellipsoid equation [19].

The prolapse was assessed using the prolapse classification system, and stage III or higher was evaluated and enrolled [20]. A urinary catheter was placed preoperatively in all patients. Each patient was administered a prophylactic intravenous cephalosporin antibiotic before the procedure.

All surgeries were performed by the first author. All participants who consented to take part in the study provided written informed consent after being fully informed about the surgical procedure, associated risks, and limitations, for both TAH and TVH. Below we described TVH and TAH techniques used in our department.

### 2.2. TVH Technique

The patient was placed in the dorsal lithotomy position, with the buttocks positioned slightly beyond the edge of the operating table. Under anesthesia, a thorough pelvic examination was performed to confirm the feasibility of the VH, including assessment of the pelvic arch, vaginal caliber, and a traction test to evaluate uterine mobility—an essential factor in selecting the vaginal approach. The operative field was then prepared with povidone–iodine applied to the vulvovaginal area. The cervix was grasped with two Tirreballe clamps to provide adequate traction, allowing clear identification of the cervicovaginal junction.

Hydrodissection was performed by infiltrating 20–50 mL of saline into the cervical area planned for incision, typically without the use of a vasoconstrictor. The anterior vaginal wall was then incised through its full epithelial thickness at the cervicovaginal junction, retracted, and the bladder was displaced caudally. After identifying the anterior peritoneal fold, it was grasped, elevated, and incised. A Breisky–Navratil speculum was used throughout this step to protect the bladder during dissection.

The posterior approach generally began with a transverse incision made between the 4 and 8 o’clock positions. The posterior vaginal edge was grasped with two Pean clamps and dissected until the posterior peritoneal fold was encountered. The peritoneum was incised with scissors, confirming entry into the posterior cul-de-sac, and a long vaginal speculum was placed for exposure.

The uterosacral ligament was clamped with a Heaney clamp, transected, and ligated, followed by the cardinal ligament, which was treated in the same manner. In most cases, both ligaments were clamped and ligated together on each side. The same sequence of steps was then performed on the contralateral side.

If anterior peritoneal access was impeded by abnormal vesicouterine adhesions, once the inferior uterine pedicles had been ligated and transected, the next set of pedicles was clamped, divided, and ligated to achieve additional uterine mobilization.

The uterine vessels were then identified and clamped between the anterior and posterior peritoneal reflections using a Heaney clamp, followed by transection and transfixion ligation. Bilateral progressive clamping of the uterine artery was carried out in a cranial direction using the same technique. After achieving complete vascular control, the uterine fundus was identified and grasped with a traction clamp. Downward and anterior pressure allowed for exteriorization of the fundus. The utero-ovarian vessels, fallopian tube, and round ligament were then double-clamped and ligated, completing uterine detachment and concluding the hysterectomy.

After uterine removal, a routine inspection of the adnexa was performed by applying lateral and downward traction on the utero-ovarian pedicle clamps. A right-angle or Breisky retractor was inserted laterally into the peritoneal cavity to enhance visualization. Once the fallopian tubes and ovaries were exposed, they were gently grasped with a Babcock clamp to facilitate adnexal traction.

Next, a Heaney clamp was placed lateral to the utero-ovarian pedicle and advanced superiorly, above the tube and ovary, to clamp the infundibulopelvic ligament and secure the gonadal vessels. A resorbable suture was then applied around the Heaney clamp, allowing for double clamping and ligation of this pedicle for added hemostatic security. A portable electrosurgical vessel-sealing device was also used to assist with adnexal excision.

Hemostasis was assessed beginning at the cervicovaginal pedicle and proceeding clockwise. To ensure adequate hemostasis, the retroperitoneal space was obliterated by suturing the anterior peritoneal fold to the anterior vaginal cuff and the posterior peritoneum to the posterior cuff. The vaginal vault was subsequently closed by approximating the anterior and posterior vaginal walls with interrupted absorbable “X” sutures. A peritoneal drain was placed in both the TVH and the TAH groups, according to our department’s standard surgical protocol. In the TVH group, the drain was introduced through the vaginal cuff into the abdominal cavity, while in the TAH group it was placed via the abdominal route. In both groups, the drain was routinely removed 48 h after surgery. Vaginal packing with povidone–iodine-soaked gauze was applied to monitor postoperative bleeding and promote hemostasis through compression. Cystourethroscopy was performed at the end of the procedure whenever bladder injury was suspected to confirm the integrity of the bladder and ureters.

### 2.3. TAH Technique

The patient was positioned in Trendelenburg after the surgical field was prepared. A vesico-urethral probe was applied with continuous monitoring of the amount and appearance of diuresis.

The abdominal approach was performed through a Pfannenstiel or Maylard incision, depending on the particularities of the case. In the case of a Maylard incision, the aponeurosis was sectioned, followed by sectioning of the rectus abdominis muscles and clamping, sectioning, and ligation of the inferior epigastric vessels.

After entering the peritoneal cavity, the median umbilical ligament (i.e., urachus) was clamped, sectioned, and ligated, its lower portion being used to facilitate exposure of the surgical field by traction of the urinary bladder. A systematic inspection of the peritoneal cavity was performed, and, when necessary, adhesiolysis was performed. Exposure was optimized by using abdominal drapes and a Gosset-type autostatic retractor, and for effective uterine traction, two Pean-type forceps were applied bilaterally to the uterine horns, encompassing the insertion of the round ligament, the utero-ovarian ligament, and the fallopian tube. The round ligaments were clamped with Kocher forceps, then sectioned and ligated bilaterally.

Further, the posterior leaf of the broad ligament was sectioned for retroperitoneal access to the lumbo-ovarian ligament. The pelvic ureter was identified, a breach was created inferior to the lumbo-ovarian ligament, after which the infundibulo-pelvic ligament was clamped with a J.L. Faure-type forceps, sectioned, and double-ligated using 2-0 Vicryl absorbable suture. The sectioning of the broad ligament sheets was continued until the vicinity of the uterus, followed by detachment of the appendix.

Then, bilateral adnexectomy was performed, followed by clamping the utero-ovarian ligament with J.L. Faure forceps, sectioning and ligating it with Vicryl 0 or 1 absorbable thread.

Operative time was measured from the initial incision to the completion of the procedure. Intraoperative blood loss was routinely estimated by the surgical nurse based on the number of soaked mops: approximately 20 mL for a quarter-soaked mop, 40 mL for a half-soaked mop, and 80 mL for a fully soaked mop. The specimen was weighed immediately after surgery. Hospital stay was defined as the total number of days the patient remained hospitalized.

### 2.4. Uterine Reduction Size Techniques

The subsequent steps following hysterectomy depended on the size and characteristics of the uterine mass. In most cases, we employed uterine hemisection, tactical myomectomy, or morcellation, as described below.

Uterine hemisection involves dividing the uterus in the sagittal plane, beginning at the cervix and proceeding cranially, while maintaining the incision within the uterine cavity to prevent lateral deviation and minimize bleeding. Once hemisection was complete, one half of the specimen could be temporarily internalized into the abdominal cavity, facilitating safe exposure and access to the contralateral side for ligation and dissection. Tactical myomectomy and morcellation were particularly used as an additional technique to sequentially excise myometrial fragments when necessary.

### 2.5. Post-Operative Follow-Up

All patients followed a standardized post-operative pain management protocol, which included two doses of intravenous meperidine (50 mg) every 4 h, followed by acetaminophen (325 mg) every 6 h. Patients were discharged once they were able to manage pain with oral medication alone, tolerate a soft diet, urinate independently, and had resumed normal bowel function.

Intra-operative complications were documented, including depolishing of a large intestinal loop and inadvertent cystostomy requiring cystorrhaphy. Post-operative complications were recorded as secondary anemia, urinary tract infection (UTI), dynamic ileus, and wound seroma.

### 2.6. Statistical Analysis

Statistical analyses were performed using IBM SPSS Statistics version 23. For continuous variables, the principal measures of central tendency and dispersion are summarized in Table 1. Categorical variables were expressed as percentages for each category in Table 2.

Normality was assessed using the Kolmogorov–Smirnov test. For hypothesis testing, the Independent Samples *t*-test, Mann–Whitney U test, and the Median test were applied, with the significance level (α) set at 0.05. The Kolmogorov–Smirnov test indicated that the normality assumption was met only for the age variable (*p* > 0.05), whereas for most categories of group variable, the assumption was violated (*p* < 0.05). Accordingly, nonparametric methods (i.e., Mann–Whitney U and Median tests) were used for the respective analyses.

## 3. Results

A total of 180 patients underwent either TVH or TAH during the study period. The common indication for both groups was for the uterus with UP and a weight between 250 and 300 g. TVH with bilateral adnexectomy was achieved in 88 patients (97.77%) and TAH with bilateral adnexectomy in 89 patients (98.88%). From TVH group, only 3 patients (3.33%), and from TAH group, only 9 patients (10.00%) had unilateral salpingectomy, which did not affect our results. The other patients had previously undergone adnexectomy. The main clinical demographics, presented using dispersion for continuous variables for the patients undergoing both TVH and TAH, can be found in Table 1. There were no statistically significant differences between the mean ages of the two groups, TVH (i.e., 66.54 ± 7.40 years) and TAH (i.e., 66.16 ± 7.31 years, t = 0.355, df = 178, *p* = 0.723 > α = 0.05).

For the uterine weight variable, the non-parametric Mann–Whitney U test indicated no statistically significant differences between the TVH group (i.e., median = 273.50, IQR = 26.25, average rank = 80.16) and the TAH group (i.e., median = 287.00, IQR = 28.75, average rank = 100.84; U = 3119.50, z = −1.665, *p* = 0.009 > α = 0.05). The Median test confirmed the absence of a statistically significant difference (i.e., Chi-Square = 3.529, df = 1, *p* = 0.060 > α = 0.05).

For the BMI variable, the Mann–Whitney U test also showed no statistically significant differences between the TVH group (i.e., median = 27.55, IQR = 8.27, average rank = 90.86) and the TAH group (i.e., median = 27.60, IQR = 8.42, average rank = 90.14; U = 4017.50, z = −0.093, *p* = 0.926 > α = 0.05). The Median test yielded similar results (i.e., Chi-Square = 0, df = 1, *p* = 1.000 > α = 0.05).

A similar pattern was observed for parity, with no statistically significant differences between the TVH group (i.e., median = 2.00, IQR = 2.00, average rank = 28.72) and the TAH group (i.e., median = 2.00, IQR = 2.00, average rank = 92.28; U = 3889.50, z = −0.477, *p* = 0.633 > α = 0.05). The Median test supported this finding (i.e., Chi-Square = 0.277, df = 1, *p* = 0.634 > α = 0.05).

**Figure 1:**
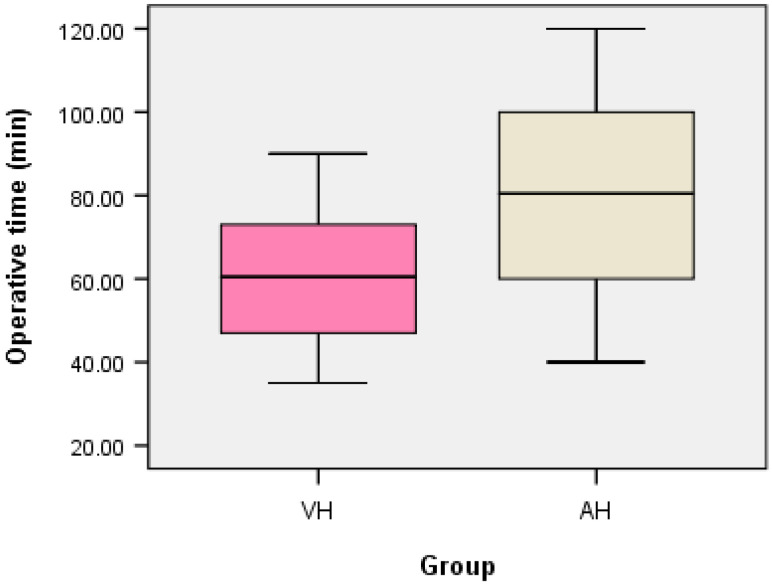
The boxplot of the operative time (minutes) for the TVH and TAH groups.

In contrast, statistically significant differences were found for operative time, with lower values in the TVH group (i.e., median = 60.50, IQR = 26.00, average rank = 68.97) compared to the TAH group (i.e., median = 80.50, IQR = 40.75, average rank = 112.03; U = 2112.00, z = −5.545, *p* < 0.001 < α = 0.05). The Median test confirmed this result (i.e., Chi-Square = 15.022, df = 1, *p* < 0.001 < α = 0.05) (Figure 1).

Similarly, for intra-operative blood loss, the Mann–Whitney U test showed statistically significant differences between the TVH group (i.e., median = 102.50, IQR = 48.50, average rank = 68.24) and the TAH group (i.e., median = 132.50, IQR = 59.00, average rank = 112.76; U = 2047.00, z = −5.254, *p* < 0.001 < α = 0.05), supported by the Median test (i.e., Chi-Square = 17.422, df = 1, *p* < 0.001 < α = 0.05).

Regarding hospital stay, statistically significant differences were also observed, with shorter hospital stays in the TVH group (i.e., median = 4.00, IQR = 2.00, average rank = 60.08) compared to the TAH group (i.e., median = 5.00, IQR = 1.00, average rank = 120.92; U = 1312.50, z = −8.084, *p* < 0.001 < α = 0.05). The Median test yielded consistent results (i.e., Chi-Square = 54.783, df = 1, *p* < 0.001 < α = 0.05).

Medical history showed to be more common in TAH group (i.e., 6.67% patients had secondary anemia, 3.33% type 2 diabetes, 8.89% hypertension, 3.33% dyslipidemia, 3.33% hypertension and type 2 diabetes, 2.22% stroke, 5.56% hypothyroidism, 2.22% chronic kidney disease, 2.22% hypertension and dyslipidemia, 1.11% asthma, 4.44% osteoporosis, 1.11% anxiety syndrome, 4.44% hypertension, type 2 diabetes and dyslipidemia and 1.11% type 2 diabetes and dyslipidemia) compared to TVH group (i.e., 3.33% had secondary anemia, 4.44% type 2 diabetes, 10.00% hypertension, 3.33% dyslipidemia, 2.22% hypertension and type 2 diabetes, 3.33% hypothyroidism, 3.33 hypertension and dyslipidemia, 1.11% asthma, 1.11% depressive syndrome, 1.11% rheumatoid arthritis, 3.33% osteoporosis, 2.22% hypertension, type 2 diabetes and dyslipidemia and 2.22% type 2 diabetes and dyslipidemia).

Surgical history showed the same results in TAH group (i.e., 5.56% had appendectomy, 4.44% cholecystectomy, 1.11% bilateral adnexectomy, 11.11% caesarian section, 1.11% appendectomy and cholecystectomy, 2.22% cystectomy, 3.33% breast lumpectomy, 1.11% mastectomy and 10.00% unilateral salpingectomy) compared with TVH group (i.e., 10.00% appendectomy, 3.33% cholecystectomy 2.22% bilateral adnexectomy, 5.56% cesarean section, 3.33% appendectomy and cholecystectomy, 4.44% breast lumpectomy, 2.22% mastectomy and 3.33% unilateral salpingectomy).

However, intra-operative complications (i.e., only 1.11% from the TVH group consisted of one case with inadvertent cystostomy–cystorrhaphy vs. 1.11% from the TAH group consisted of one case with depolishing of a large intestinal loop) and post-operative complications (i.e., 3.33% from the TVH group consisted of one patient with anemia, and two patients with UTI vs. 9.99% from the TAH group consisted of four patients with anemia, two patients with dynamic ileus, and three patients with wound seroma) were slightly more frequent in the TAH group. The operation was successfully completed in all patients with a planned TVH, without requiring any conversion at laparotomy or laparoscopy (Table 2).

**Figure 2:**
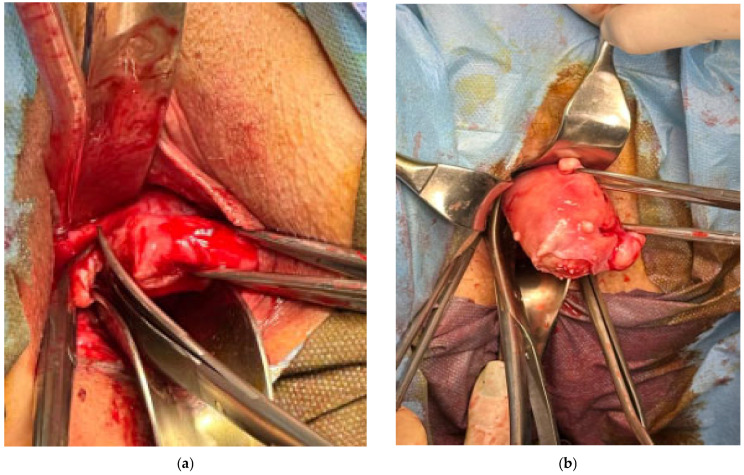
The TVH technique: (**a**) clamping and sectioning of the right inferior pedicle; (**b**) clamping and sectioning of the right superior pedicle.

Adhesions were more common in TAH cases (i.e., 33.33% for the TAH group vs. 13.33% for the TVH group). Uterine hemisection, tactical myomectomy, and morcellation were used in most cases (i.e., *n* = 73, 81.11%) to obtain a reduction in uterine size in the TVH group, whereas intramyometrial reduction and morcellation after cervical amputation were used only in a few cases (i.e., *n* = 17, 18.88%, Figure 2).

## 4. Discussion

### 4.1. Patients’ Outcomes

In the present study, post-operative complications were slightly more frequent in the TAH group (i.e., 9.99% vs. 3.33%), similar to other studies, possibly due to different socio-economic or nutritional status [21,22,23]. Further, in our study, uterine hemisection, tactical myomectomy, or morcellation were used in most cases to obtain a reduction in uterine size for the TVH group, which was in accordance with other studies [24,25,26].

However, TVH is used more for UP (i.e., approximately in 10 till 66.7% cases), and the route of hysterectomy is mostly dependent on the institutional guidelines, personal preference, or the experience of the surgeon [6]. Today, still, a small number of surgeons are competent in performing different hysterectomy types, but most are comfortable with one route only [27]. Some characteristics, like the uterine size, pelvic pathology, pelvic surgery, BMI, parity, or need for oophorectomy, could also become evidence of implementation of various practices, expanding in this way the list of indications for TVH [28].

In our study, the enlarged uterus was chosen, knowing its definition, which relies on approximately 280 g [29,30]. VH for the enlarged uterus brings about prolonged operation time, increased blood loss, and the risk of retroperitoneal bleeding [31].

Our study highlights the fact that VH can be employed to remove uteri of more than 250 g. Though the current ACOG guidelines do not recommend the use of VH over TAH in such cases, the results from our data and other retrospective studies further confirm that VH is associated with better outcomes [32,33] compared to the abdominal approach for the larger uteri. Our findings are consistent with the randomized data from Benassi et al. [34], who found that VH (*n* = 60) compared to TAH (*n* = 59) for the uterus between 200 g and 1300 g was associated with significantly longer operative times but shorter hospital stay, without an increase in major complication rates. There are several strategies that surgeons can use to reduce uterine size in order to facilitate VH. In the study of Fatania et al. [35], 46% of patients in the VH group received pre-operative GnRH, and for 84.6% of patients whose uterus was removed vaginally, surgeons utilized techniques such as morcellation, wedge resection, or bisection. Medically debulking the uterus, using GnRH pre-operatively, has been shown to reduce its size, thereby increasing the chance of delivering it safely during VH [36]. More women had GnRH analogs to shrink the uterus in order to facilitate VH, but the uterine sizes at the time of surgery were similar (i.e., 403.1 g for TVH vs. 460.5 g for TAH). However, in our study, GnRH was not used to reduce the size of the uterus.

Therefore, no significant differences were found between groups in terms of mean age, uterine weight, BMI, or parity. TVH was associated with significantly shorter operative time, lower intra-operative blood loss, and reduced hospital stay compared to TAH (*p* < 0.001). Interestingly, there was not found any wound infection, being associated at the same time with less bleeding requiring transfusion and re-admission for the TVH group. Our results are in agreement with other studies, and this could be due to the fact that all hysterectomies were conducted by a well-trained gynecologist surgeon, along with the importance of using ultrasonography for a proper diagnosis [37].

In our study, a successful, complication-free VH offers a highly effective, efficient, and patient-friendly solution for UP, aligning with goals for quick recovery, minimal impact on daily life, and good functional outcomes, showing its practical significance for patients with UP and an enlarged uterus of 250–300 g. Our study is in agreement with the study of Pakbaz and contributors, which found that hysterectomy performed for vaginal prolapse in a routine clinical health care setting results in a short hospital stay, swift recovery, and a low rate of complications with a high grade of patient satisfaction [38]. Further, some authors also consider that this technique should be indicated to treat gynecological benign disease, having fewer intra- and post-operatively complications, shorter hospital stay, and low morbidity on the uterus with a weight of 278.9 g [39]. Another study sustains the hypothesis that a very large uterine volume does not represent a real obstacle to perform TVH and that results in a safe and effective technique in cases of uterine weight ≥ 250 g [40]. Another study showed that TVH can be performed on a uterus weighing a maximum of 540 g; however, techniques for vaginal morcellation must also be mastered. Authors conclude the VH advantages as the first-choice method, which was possible in two-thirds of the analyzed cases without stretching the limits of the method [41].

VH is still considered a top-tier minimally invasive approach, often preferred over laparoscopic or robotic methods for appropriate cases due to faster recovery, less pain, and lower cost. The technique gives some advantages for complex situations like benign conditions, with robotics having comparable outcomes, but higher costs for simple cases [42].

Recent efforts of societies, such as The American College of Obstetricians and Gynecologists [43] and the American Association of Gynecologic Laparoscopists [44], have verbalized that VH should still be considered the approach of choice in the treatment of benign diseases whenever feasible, and should be the preferred therapeutic strategy among all minimally invasive approaches. However, in the case of operating time, intra- or post-operative complications, VH seems to be the fastest approach when compared to robotics and laparoscopy [42].

The variability may be attributed to differences in health care systems among the countries where the studies were conducted, as well as to the progressive evolution of perioperative management. Over time, a gradual transition from laparotomic to laparoscopic approaches has been observed.

Reduced blood loss, shorter hospital stays, and faster recovery of bowel function represent the main advantages of minimally invasive surgery; however, complication rates are comparable to those of open abdominal procedures. Although minimally invasive techniques like VH may provide meaningful benefits in perioperative outcomes, their impact on long-term prognosis has yet to be clearly established [45].

Nevertheless, laparoscopic surgery offers several advantages compared with open procedures. Key aspects of post-operative management include effective pain control, early mobilization, close monitoring using early warning scores, and the provision of appropriate discharge instructions [46].

### 4.2. Strengths and Weaknesses of the Study

One of the strengths of our study could be represented by the homogenous population and uniformity in the surgical techniques achieved by the same surgeon. Moreover, the study describes the TVH technique in a way that facilitates its reproduction. Therefore, our study showed a significant reduction in operative time, blood loss, and length of hospital stay for TVH, which underlines the economic and logistical advantages of the vaginal approach, a relevant aspect for resource-limited health care systems. Finally, another strength of our study could be explained by the fact that TVH has more advantages than TAH, as none of our cases needed conversion to TAH, and the data from a tertiary center, reflecting real experience in clinical practice.

The weaknesses of our study could be represented by the lack of randomized studies, retrospective design, possible confounding, or surgeon bias. However, the weaknesses also involve limitations to a larger uterus or certain conditions, such as a small number of patients with previous surgeries causing extensive adhesions. More important is the fact that decisions to perform a hysterectomy were made by individual surgeons, without any additional prolapse surgery (i.e., cystocele or rectocele). Long-term effects on pelvic function, recurrence of UP, or quality of life were not the main aim of our study. In some cases, the possibility of more initial pain than the laparoscopic technique exists, or specific medical conditions that require better visual access to the pelvic area, adnexectomy challenges, and surgeon skill dependence, although VH is generally a preferred method for benign conditions when feasible.

## 5. Conclusions

The route of hysterectomy did not influence morbidity, but had an influence on the operative time, intra-operative blood loss, and duration of hospital stay related to using TVH for the uterus with a weight between 250 and 300 g with UP. The present study further supports the idea that all patients requiring hysterectomy for such conditions can be offered TVH, which could become a better therapy option.

## Figures and Tables

**Table 1:** Measures of central tendency and dispersion for the continuous variables in the TVH and TAH groups.

Characteristics	TVH Group (*n* = 90)	TAH Group (*n* = 90)
Uterine weight (g)		
Mean	275.00	280.89
SD	15.12	15.43
Median	273.50	287.00
IQR	24.50	26.25
Body mass index (kg/m^2^)		
Mean	27.87	25.83
SD	4.80	4.89
Median	27.55	27.60
IQR	8.27	8.42
Parity		
Mean	2.21	2.33
SD	1.29	1.38
Median	2.00	2.00
IQR	2.00	2.00
Operative time (min)		
Mean	60.52	80.09
SD	15.16	23.46
Median	60.50	80.50
IQR	26.00	40.25
Intra-operative blood loss (mL)		
Mean	101.46	134.40
SD	28.68	35.62
Median	102.50	132.50
IQR	48.50	59.00
Hospital stay (days)		
Mean	3.99	5.67
SD	0.80	1.46
Median	4.00	5.00
IQR	2.00	1.00

**Table 2:** Observed frequencies and corresponding percentages of the categorical variables in the TVH and TAH groups.

Characteristics	TVH Group, *N* (%)	TAH Group, *N* (%)
Medical history	37 (41.11)	45 (50.00)
Surgical history	31 (34.44)	36 (40.00)
Intra-operative complications	1 (1.10)	1 (1.10)
Post-operative complications	3 (3.33)	9 (9.99)
Adhesions	12 (13.33)	30 (33.33)

## Data Availability

All data supporting the findings of this study are included within the article.

## Abbreviations

The following abbreviations are used in this manuscript:

TVH	Total vaginal hysterectomy
TAH	Total abdominal hysterectomy
UP	Uterine prolapse
BMI	Body mass index
UTI	Urinary tract infection
SD	Standard deviation
IQR	Interquartile range
